# Temporal muscle thickness is an independent prognostic marker in melanoma patients with newly diagnosed brain metastases

**DOI:** 10.1007/s11060-018-2948-8

**Published:** 2018-07-14

**Authors:** Julia Furtner, Anna S. Berghoff, Veronika Schöpf, Robert Reumann, Benjamin Pascher, Ramona Woitek, Ulrika Asenbaum, Sebastian Pelster, Johannes Leitner, Georg Widhalm, Brigitte Gatterbauer, Karin Dieckmann, Christoph Höller, Daniela Prayer, Matthias Preusser

**Affiliations:** 1 Department of Biomedical Imaging and Image-guided Therapy, Medical University of Vienna, Waehringer Guertel 18-20, 1090 Vienna, Austria; 2 Comprehensive Cancer Center, Medical University of Vienna, Waehringer Guertel 18-20, 1090 Vienna, Austria; 3 Department of Medicine I, Medical University of Vienna, Waehringer Guertel 18-20, 1090 Vienna, Austria; 4 Institute of Psychology, University of Graz, Universitaetsplatz 2, 8010 Graz, Austria; 5 BioTechMed, Mozartgasse 12, 8010 Graz, Austria; 6 Department of Neurosurgery, Medical University of Vienna, Waehringer Guertel 18-20, 1090 Vienna, Austria; 7 Department of Radiotherapy, Medical University of Vienna, Waehringer Guertel 18-20, 1090 Vienna, Austria; 8 Department of Dermatology, Medical University of Vienna, Waehringer Guertel 18-20, 1090 Vienna, Austria

**Keywords:** Melanoma, Neoplasm metastasis, Brain, Sarcopenia, Prognosis

## Abstract

**Objectives:**

The purpose of this study was to evaluate the prognostic relevance of temporal muscle thickness (TMT) in melanoma patients with newly diagnosed brain metastases.

**Methods:**

TMT was retrospectively assessed in 146 melanoma patients with newly diagnosed brain metastases on cranial magnetic resonance images. Chart review was used to retrieve clinical parameters, including disease-specific graded prognostic assessment (DS-GPA) and survival times.

**Results:**

Patients with a TMT > median showed a statistically significant increase in survival time (13 months) compared to patients with a TMT < median (5 months; p < 0.001; log rank test). A Cox regression model revealed that the risk of death was increased by 27.9% with every millimeter reduction in TMT. In the multivariate analysis, TMT (HR 0.724; 95% 0.642–0.816; < 0.001) and DS-GPA (HR 1.214; 95% CI 1.023–1.439; p = 0.026) showed a statistically significant correlation with overall survival.

**Conclusion:**

TMT is an independent predictor of survival in melanoma patients with brain metastases. This parameter may aid in patient selection for clinical trials or to the choice of different treatment options based on the determination of frail patient populations.

## Introduction

Melanoma patients have the highest risk of developing brain metastases during their course of disease, with an incidence of up to 70%, ahead of breast or lung cancer patients [1, 2].

The presence of brain metastases results in a devastating impairment in the quality of life and is associated with reduced survival times. The median overall survival (OS) is 4–6 months in unselected melanoma patients with brain metastases and 7–10 months in case of stereotactic radiosurgery or surgery [3–6]. Targeted therapies have opened up a new horizon in the treatment of metastatic melanoma patients [7]. However, therapy selection depends on several various factors, including histological and molecular tumor characteristics, size, location, and number of intracranial lesions, extracranial disease spread, as well as the patient’s overall physical condition. While some of these parameters are objectively measurable, others, such as the assessment of the clinical condition of the patient, are limited by high observer variability because they are mainly based on the subjective evaluation of the attending physician [8]. In particular, these scores lack accuracy in predicting survival in all cancer patients [9]. Therefore, objective parameters to assess a patient’s clinical condition are urgently needed to improve outcome prediction. A recently established objective parameter by which to define the patient’s frailty is the determination of skeletal muscle mass. The loss of skeletal muscle mass and function is referred to as sarcopenia, which is a main condition of cancer-related cachexia [10]. Previously published studies have indicated a high association between sarcopenia and long-term outcome in various cancer entities [11–14]. A well established technique to determine sarcopenia is based on sex-dependent skeletal muscle mass index values obtained by abdominal computed tomography images at the level of L3 [11, 14, 15]. Recently, an association between skeletal muscle mass and temporal muscle thickness (TMT) has been shown [16]. TMT can be assessed easily on routine magnetic resonance imaging (MRI) examinations and implies that craniofacial muscles are valuable indicators with which to predict patient frailty, which would be especially advantageous in patients with intracranial neoplastic lesions.

The purpose of this study was to evaluate the prognostic value of TMT in melanoma patients with brain metastases to explore the importance of TMT as a surrogate marker of patient frailty.

## Methods

### Patients

One hundred forty-six melanoma patients with newly diagnosed brain metastases between 2002 and 2014 were retrieved from the brain metastasis database of the Medical University of Vienna. The following were the inclusion criteria: (a) available MRI examination of the brain; (b) at least on one side, the temporal muscle had to be depicted in its whole extension without any signs of previous intervention that would have affected the muscle thickness (e.g., previous craniectomy with muscle edema or subsequent muscle atrophy); and (c) information available about the weight and height of the patients to calculate the body mass index (BMI) within 1 month before or after the MR examination. Chart review was used to retrieve further clinical information regarding the clinical course, including diagnosis-specific graded prognostic assessment (DS-GPA) at the time of brain metastases diagnosis, as well as survival times. DS-GPA is a well established and validated prognostic assessment based on various clinical factors depending on the underlying primary tumor type. In melanoma patients, the DS-GPA is based on the number of brain metastases and the Karnofsky performance score and was applied as previously outlined by Sperduto et al. [17].

For the present study, OS was defined as days between the diagnosis of brain metastasis and death or date of last follow-up.

The study was approved by the ethics committee of the Medical University of Vienna (Vote 078/2004).

### TMT assessment

**Fig. 1:**
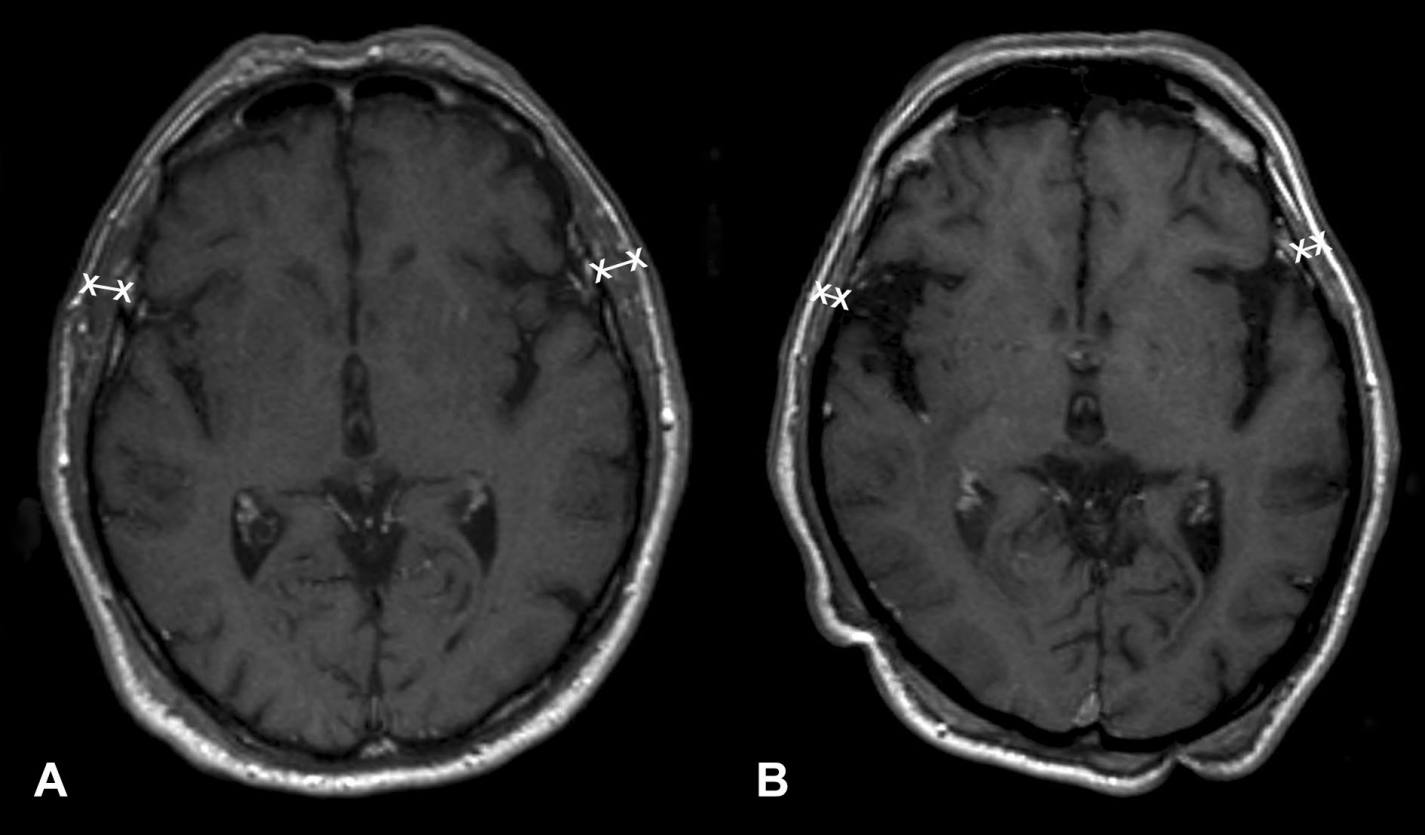
TMT assessment represented on brain MR images. **a** A 77-year-old male patient with an overall survival of 15 months (median TMT = 8.8 mm), and **b** a 73-year-old male patient with an overall survival of 3 month (median TMT = 2.8 mm)

Examples of TMT measurements on MR images of the brain are shown in Fig. 1.

TMT was assessed at the time of diagnosis of brain metastases on axial, isovoxel (1 × 1 × 1 mm^3^) T1-weighted MR images. The plane was oriented parallel to the anterior commissure–posterior commissure line. The measurements were taken perpendicular to the long axis of the temporal muscle using the orbital roof (cranio-caudal) and the Sylvian fissure (anterior–posterior) as anatomical landmarks. TMT was measured on the left and on the right side separately in each patient by a board-certified radiologist (JF). TMT of each side was summed up and divided by two, resulting in a mean TMT per patient.

### Statistical analysis

The median TMT of all patients was calculated to divide the patient cohort in two groups and apply the Kaplan Meier product to calculate survival curves. To determine the differences between those two groups, a log-rank test was used. A Cox regression model was used to evaluate the association between OS and TMT as a scale variable, as well as to calculate the association with survival times in a multivariate analysis, including the well established DS-GPA.

The strength of the association between two scale variables was calculated using a Spearman correlation coefficient. This correlation coefficient was classified as very strong (± 0.8–1), strong (± 0.6–0.8), moderate (± 0.4–0.6), low (± 0.2–0.4), or nonexistent (± 0–0.2).

A two-tailed p value of < 0.05 was defined as statistically significant. Statistical analysis was performed using SPSS Version 24.0.

## Results

The study cohort consisted of 146 melanoma patients with newly diagnosed brain metastases. An overview of patient characteristics is given in Table 1.

**Table 1:** Patient characteristics

	n	%
Median age at diagnosis of brain metastases, years (range)	60 (23–88)	
Gender		
Male	96	65.8
Female	50	34.2
Median BMI (range)	26.1 (16.2–40.8)	
1st line treatment after BM diagnosis		
Stereotactic radiosurgery	82	56.2
Chemotherapy	2	1.4
Neurosurgical resection	43	19.5
Whole-brain radiotherapy	17	11.6
Best supportive care	2	1.4
Diagnosis-specific GPA		
0–1.0	17	11.6
1.5–2.0	36	24.7
2.5–3.0	43	29.5
3.5–4.0	50	34.2
Alive at last follow-up		
Yes	10	6.8
No	136	93.2
Median overall survival from diagnosis of BM, months (range)	7.6 (0–84)	

The mean TMT of female patients was 5.0 mm (2–8.9), and 6.2 mm (1.7–10.8) in male patients, resulting in an overall mean TMT of 5.8 mm (range 1.7–10.8). Male patients showed significantly higher mean TMT values (6.2 mm) compared to female patients (5.0 mm) (p < 0.001; Mann–Whitney-*U* test). With regard to other clinical parameters, mean TMT showed a low negative association with patient age at the diagnosis of brain metastases (Spearman correlation coefficient − 0.231; p = 0.005) and no correlation with patient BMI (Spearman correlation coefficient 0.097; p = 0.245).

**Fig. 2:**
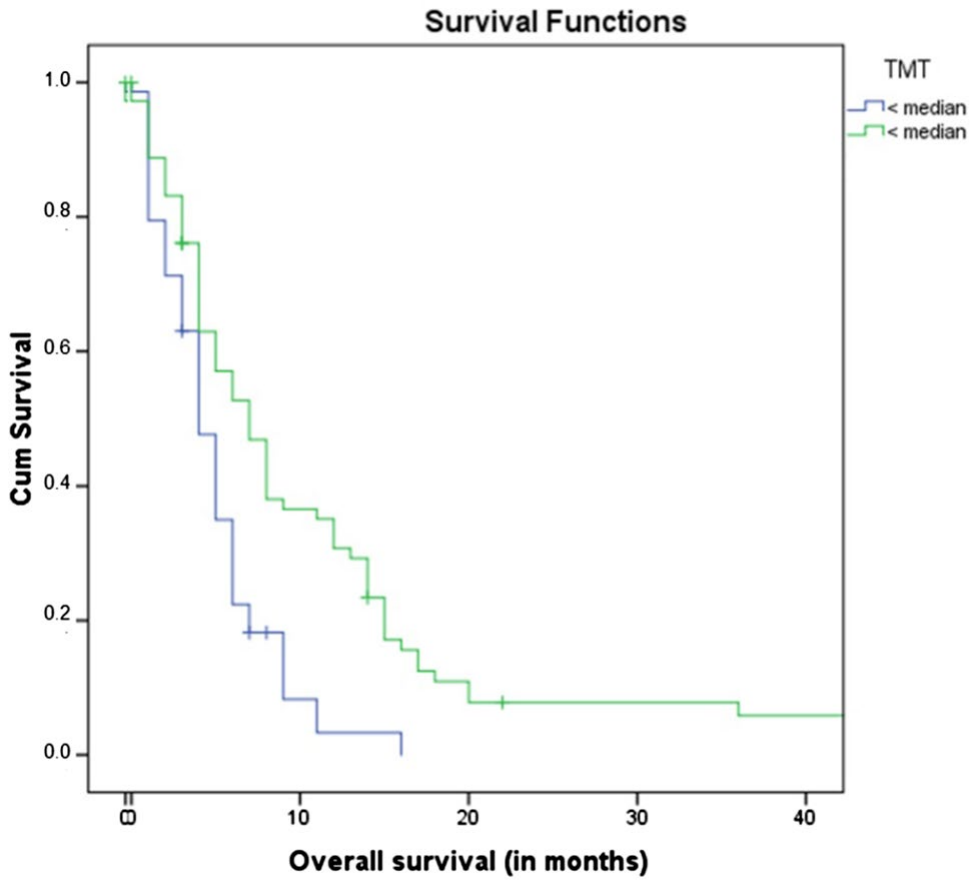
Overall survival according to median TMT

Survival analysis was performed using a Cox regression model with TMT diameters to predict survival time. Patients with a TMT above the median had a significantly improved survival prognosis, with a hazard ratio (HR) of 0.721 (95% CI 0.642–0.810; p < 0.001; Cox regression model) compared to patients with TMT below the median. In detail, the risk of death will increase by 27.9% with every millimeter reduction in TMT. Patients with a TMT > median showed a statistically significant increase in survival time (13 months) compared to patients with a TMT < median (5 months; < 0.001; log-rank test; Fig. 2).

To investigate the association between survival prognosis and different covariates, including TMT and DS-GPA, further analysis was performed with a Cox regression model. In the multivariate model, TMT (HR 0.724; 95% 0.642–0.816; < 0.001) and DS-GPA (HR 1.214; 95% CI 1.023–1.439; p = 0.026) showed a statistically significant correlation with OS. Explicitly, the TMT prediction value of survival was nearly unchanged, with an increased risk of death of 27.6% with every millimeter reduction in TMT. Adding patient sex to the multivariate model in the same fashion did not explain additional variance (p = 0.091).

## Discussion

In this study, the prognostic role of TMT in melanoma patients with brain metastases was investigated. A clearly defined patient cohort was selected, which consisted of melanoma patients with newly diagnosed brain metastases, an available MRI of the brain at the diagnosis of brain metastases, and a full clinical follow-up. We could show a strong correlation between TMT and patient survival. In detail, the risk of death was reduced by 27.9% with each millimeter of TMT, independently of the established prognostic DS-GPA score. Therefore, TMT is an objectively determinable marker that can help to improve the prognosis of melanoma patients with brain metastases. The results are in line with recently published data about breast cancer and non-small lung cancer patients with brain metastases [18]. In those studies, TMT was found to reduce the risk of death by 19 and 24% in breast cancer and non-small cell lung cancer patients, respectively, at the diagnosis of brain metastases.

Furthermore, TMT measurements were correlated with different clinical parameters in order to ensure that the measurements were not influenced by other patient characteristics. The findings are in accordance with previously published data [18]. No association was found between TMT and BMI. This is based on the fact that TMT represents skeletal muscle mass while BMI focuses only on the patient’s weight with no regard to body composition. Therefore, BMI is not able to identify sarcopenia in an obese patient population. Moreover, only a weak negative correlation was identified between TMT and patient age, indicating that, in terms of survival prediction, the patient’s physical condition reveals more information than the patient’s age. Although TMT values were significantly lower in female patients compared to male patients, the prognostic value of TMT measurements was valid, independent of patient sex.

The findings of this study are consistent with the present literature, as skeletal muscle mass has been revealed to be a prognostic factor in various diseases, including different cancer types. This is based on the fact that muscle wasting is associated with cancer-related cachexia, which is known to have a multi-factorial etiology and is not reversible based solely on nutrition. Therefore, it is all the more important to assess the skeletal muscle mass routinely in cancer patients to be able to delay the progression or even improve muscle mass loss using new therapeutic strategies, including physical training, specific medications, or nutritional supplements [19–22].

The temporal muscle has been shown previously to have potential for outcome prediction. Lisiecki et al. revealed an association between TMT and hospital-based clinical outcome parameters, such as ventilator and hospital days, in a trauma patient cohort [23]. Moreover, Rinkinen et al. investigated an inverse correlation of temporal muscle volume and hospital stay in children with nonsyndromic craniosynostosis [24]. In addition, other cranio-facial muscles have been used to assess skeletal muscle mass loss; however, on routine brain MR images, those muscles were, in most cases, only depicted incompletely [25]. Therefore, we chose the temporal muscle to investigate outcome prediction, which is a fast and easily assessable marker, and moreover, showed an excellent inter-rater reliability in a previous study [18].

A potential limitation of this study is that TMT might be influenced by oral or dental diseases [26]. Therefore, we measured TMT values on both sides and calculated the mean TMT value for each patient to reduce dental- or oral-related muscle changes as much as possible. To avoid interventions that could affect the thickness of the temporal muscle leading to subsequent muscle edema or atrophy, such as craniectomy or radiotherapy, we assessed TMT at the time of diagnosis of brain metastases. Furthermore, the retrospective design of this study excluded the possibility for anatomical–functional relationships. Therefore, the results of this study should be validated in a prospective setting with additional muscular strength correlation or other clinical frailty parameters. Moreover, a prospective investigation should focus on the correlation between TMT and skeletal muscle mass index obtained by abdominal computed tomography images at the level of L3, which is a well established method to determine sarcopenia [11, 14, 15]. A disadvantage of using TMT as a predictive marker in melanoma patients is that the assessed diameter of the temporal muscle is relatively small. It is, therefore, all the more important to adhere strictly to the predefined anatomical landmarks and take precise measurements on enlarged MR images in order to increase measurement accuracy. Other studies used temporal muscle volume for outcome prediction [24]. However, manual volume or plane segmentation of structures is time-consuming, automatic tissue segmentation usually relies on improved software tools, is still mostly prone to errors, and dependent on additional manual corrections. TMT assessment has been shown to have an excellent inter-rater reliability in a previous study and the TMT measurement per patient took only approximately 30 s [18]. Therefore, we feel that TMT is a suitable value to be integrated into the clinical workflow.

We conclude that TMT is a useful marker for survival prediction in melanoma patients with brain metastases. Assessment of TMT in the clinical setting may help in the decision about treatment options and patient stratification for clinical trials in order to define a frail patient population.

## Abbreviations

BMI: Body mass index
CT: Computed tomography
DS-GPA: Diagnosis-specific graded prognostic assessment
MRI: Magnetic resonance imaging
OS: Overall survival
TMT: Temporal muscle thickness
